# High PD-L1 Expression Correlates with Metastasis and Poor Prognosis in Oral Squamous Cell Carcinoma

**DOI:** 10.1371/journal.pone.0142656

**Published:** 2015-11-12

**Authors:** Yueh-Min Lin, Wen-Wei Sung, Ming-Ju Hsieh, Shih-Chen Tsai, Hung-Wen Lai, Shu-Mei Yang, Ko-Hong Shen, Mu-Kuan Chen, Huei Lee, Kun-Tu Yeh, Chih-Jung Chen

**Affiliations:** 1 Department of Surgical Pathology, Changhua Christian Hospital, Changhua, Taiwan; 2 School of Medicine, Chung Shan Medical University, Taichung, Taiwan; 3 Department of Medical Technology, Jen-Teh Junior College of Medicine, Nursing and Management, Miaoli, Taiwan; 4 Department of Medical Education, Chung Shan Medical University Hospital, Taichung, Taiwan; 5 Cancer Research Center, Changhua Christian Hospital, Changhua, Taiwan; 6 School of Optometry, Chung Shan Medical University, Taichung, Taiwan; 7 Institute of Medicine, Chung Shan Medical University, Taichung, Taiwan; 8 Division of General Surgery, Changhua Christian Hospital, Changhua, Taiwan; 9 School of Medicine, National Yang Ming University, Taipei, Taiwan; 10 School of Public Health, College of Public Health and Nutrition, Taipei Medical University, Taipei, Taiwan; 11 Department of Otorhinolaryngology, Head and Neck Surgery, Changhua Christian Hospital, Changhua, Taiwan; 12 Graduate Institute of Cancer Biology and Drug Discovery, Taipei Medical University, Taipei, Taiwan; Sapporo Medical University, JAPAN

## Abstract

PD-L1 has been widely demonstrated to contribute to failed antitumor immunity. Blockade of PD-L1 with monoclonal antibody could modulate the tumor immune environment to augment immunotherapy. PD-L1 expression is also detected in several types of cancer and is associated with poor prognosis. However, the prognostic role of PD-L1 in oral squamous cell carcinoma (OSCC) is still controversial. Our aim was to determine the role of PD-L1 in the prognosis of OSCC patients to identify its potential therapeutic relevance. PD-L1 immunoreactivity was analyzed by immunohistochemistry in 305 cancer specimens from primary OSCC patients. The medium follow-up time after surgery was 3.8 years (range from 0.1 to 11.1 years). The prognostic value of PD-L1 on overall survival was determined by Kaplan-Meier analysis and Cox proportional hazard models. Higher PD-L1 expression is more likely in tumor tissues of female than male OSCC patients (P = 0.0062). Patients with distant metastasis also had high PD-L1 expression (P = 0.0103). Multivariate analysis identified high PD-L1 expression as an independent risk factor in males and smokers (males: hazard ratio = 1.556, P = 0.0077; smokers: hazard ratio = 2.058, P = 0.0004). We suggest that PD-L1 expression, determined by IHC staining, could be an independent prognostic marker for OSCC patients who are male or who have a smoking habit.

## Introduction

Oral squamous cell carcinoma (OSCC) accounts for more than 550,000 cases annually worldwide and is currently the one of the leading causes of cancer-related death.[1,2] Advances have been made in both diagnosis and therapy in recent decades, and yet the prognosis of OSCC remains poor and the mortality rates are still approximately fifty percent.[3,4]. The high mortality rate could be attributed to late diagnosis and lack of specific biomarkers for predicting tumor progression and patient prognosis [5,6]. Therefore, identification of specific biomarkers would help in clinical decision making and early prediction of prognosis in OSCC.

Cancer and the immune system are fundamentally interrelated as tumors are potentially immunogenic [7]. The interactions between cancer cells and host immune cells in the tumor microenvironment create an immunosuppressive network that promotes tumor growth and protects the tumor from immune attack [7]. Several molecular mechanisms are involved in the regulation of tumor microenvironment: one of the most important is the B7 secondary signaling pathway that regulates the balance between immune potency and suppression of tumor progression [8]. The B7 family members could contribute to both antitumor immunity and tumor surveillance [8].

A role for B7 in antitumor immunity was demonstrated by the enhanced eradication of murine malignancies by cytotoxic T cells transfected to express B7-1 and B7-2 [8,9]. Similarly, promotion of tumor surveillance has been demonstrated by binding of the PD-L1 molecule (PD-L1) (also known as B7-H; B7H1; CD274; PDCD1L1; PDCD1LG1) to PDCD1 (programmed cell death 1, also known as PD1; PD-1; CD279; SLEB2; hPD-1; hPD-l; hSLE1), which generates inhibitory signals that regulate the balance among T-cell activation, tolerance, and the tumor microenvironment [10].

The PD-L1 engagement induces down-regulation of antigen-stimulated lymphocyte proliferation and ultimately results in lymphocyte exhaustion and in the induction of immunological tolerance [11,12,13]. Some studies concluded that PD-L1 expression is up regulated in solid tumors, where it can provide direct tumor protection and reduce activity of PDCD1 expressing, tumor-infiltrating effector CD4 and CD8 T cells [14,15]. Expression of PD-L1 has been reported in tumor cells of different types of cancer, including glioblastoma, ovarian cancer, renal cell carcinomas, squamous cell carcinoma of the head and neck, colon cancer, breast infiltrating ductal carcinoma, esophageal cancer, non-small cell lung cancers and melanoma [6,8,12,15,16,17,18,19,20]. A strong correlation between expression of PD-L1 on tumor cells and severe prognosis has been observed in esophageal cancer, renal cell carcinoma and lung adenocarcinoma [17,18,19,21,22,23].

The prognostic value of PD-L1 positivity in other malignancies, however, is inconsistent: Most studies reveal a worse outcome correlation [17,21,23,24], whereas favorable outcome has been observed in PD-L1 positive cancers in melanoma and colon cancer [25,26]. These conflicting results led us to investigate the role of PD-L1 in our OSCC patient population.

Information on the prevalence and prognostic role of PD-L1 expression in OSCC is limited, so we evaluated the expression and clinical significance of PD-L1 in OSCC tumors. We also investigated the prognostic role of PD-L1 in surgically resected OSCC patients according to their clinicopathological parameters.

## Materials and Methods

### Ethics Statement

This study was approved by the Institutional Review Board and the Ethics Committee of the Changhua Christian Hospital, Changhua, Taiwan (IRB no. 111014). Since the specimens were collected between 2000 and 2007, the Institutional Review Board waived the need for consent.

### Study Subjects

This study enrolled 305 OSCC patients. OSCC tumor tissues were collected between 2000 and 2007 at Changhua Christian Hospital from patients who had confirmed histological diagnosis. Cancers were staged according to seventh edition of AJCC Cancer Staging Manual. Clinical data, including smoking, alcohol consumption, betel quid chewing, gender, age, tumor stage, and T, N, and M stages, and follow-up information were obtained from medical records and the cancer registry.

### Immunohistochemistry Staining and Evaluation of PD-L1 Immunoreactivity

Immunohistochemistry (IHC) staining was performed at the Department of Surgical Pathology, Changhua Christian Hospital on tissue microarray sections (4 μm) of formalin-fixed, paraffin-embedded, pre-chemotherapy primary oral tumors using anti-human PD-L1 antibody (1:100 dilution; GTX104763, GeneTex), as previously described [27,28,29]. Antigen retrieval was performed in 95°C for 30 minutes. PD-L1 IHC was also validated. In brief, a normal tonsil was used as a positive control for the PD-L1 antibody to determine whether this antibody stains the crypts of the tonsils since this area of normal tonsil expresses endogenous PD-L1 (S1 Fig). Moreover, representative PD-L1 expression in non-neoplastic squamous epithelium was also shown in S1 Fig. When assessing PD-L1 status, cytoplasmic and membranous positivity represented a signal of interest; nuclear and extracellular staining was disregarded. Immunoreactivity scores were analyzed independently by two pathologists (YM Lin and CJ Chen), who independently scored coded sections based on the staining score without knowledge of clinical and follow-up information. Immunoreactivity scores were defined as the cell staining intensity (0 = nil; 1 = weak; 2 = moderate; and 3 = strong). PD-L1 low expression was defined as staining intensity score 0 and 1; high expression was defined as score 2 and 3. A final agreement was obtained for each score by using a multiheaded microscope (Olympus BX51 10 headed microscopes).

### Statistical Analysis

Statistical analysis was performed as previously described [30,31]. The χ^2^ test was applied for continuous or discrete data analysis. The associations between PD-L1 expression and patient survival were estimated using the Kaplan–Meier method and assessed using the log-rank test. Potential confounders were adjusted by Cox regression models, with the PD-L1 fitted as indicator variables. Gender, smoking, stage and grade were adjusted in multivariate analysis. Overall survival time was defined as the interval between the date of surgery and the date of last follow-up or death. The medium follow-up time after surgery was 3.8 years (range from 0.1 to 11.1 years). All statistical analyses were conducted using the SPSS statistical software program (version 15.0) (SPSS, Inc., Chicago, IL). All statistical tests were 2-sided, and the values of P<0.050 were considered statistically significant.

## Results

### High PD-L1 expression levels are more likely in female than male OSCC patients

We verified the PD-L1 expression in patients by recruiting 305 patients with primary OSCC tumors. The clinicopathological characteristics according to gender are listed in Table 1. As reported previously, the habits of smoking, alcohol consumption, and betel quid chewing are risk factors for OSCC. However, the percentages of female patients with these risk factors were significantly low (smoking, alcohol consumption, and betel quid chewing in female vs. male: 14.5% vs 64.8%, 10.1% vs. 54.7%, and 10.3% vs 50.9%; p values all <0.0001, Table 1). Advanced tumors were also more prevalent than early tumors in male patients (male vs. female, 59.8% vs. 33.3% for stage IV, P<0.0001; 69.1% vs. 46.4% for stage III+IV, P = 0.0006; 53.8% vs. 27.5% for T3+T4, P = 0.0001; Table 1). The N value, distant metastasis, LN metastasis, and grade of the OSCC were not significantly associated between genders. In our population, the overall survival data showed that death was a more common outcome in male than in female OSCC patients (62.3% for male vs. 24.6% for female, P<0.0001; Table 1) during our survey. High PD-L1 cytoplasm intensity was more likely in tumors from female than from male patients (staining intensity: 0–1, 60.6% for male vs. 42.0% for female; staining intensity: 2–3, 58.0% for female vs. 39.4% for male, P = 0.0062). High expression levels of PD-L1 were also more likely to occur in tumors from female than from male patients.

**Table 1:** The clinicopathological characteristics of different gender in 305 OSCC patients.

	Gender		
Parameter	Female(%)	Male (%)	P value
Smoking			
No	59(41.5)	83(58.5)	<0.0001
Yes	10(6.1)	153(93.9)	
Alcohol Consumption			
No	62(37.1)	105(62.9)	<0.0001
Yes	7(5.2)	127(94.8)	
Betel quid chewing			
No	61(37.0)	104(63.0)	<0.0001
Yes	7(6.1)	108(93.9)	
Stage			
I	29(46.8)	33(53.2)	<0.0001
II	8(16.7)	40(83.3)	
III	9(29.0)	22(71.0)	
IV	23(14.0)	141(86.0)	
Stage			
I+II	37(33.6)	73(66.4)	0.0006
III+IV	32(16.4)	163(83.6)	
T value			
T1+T2	50(31.4)	109(68.6)	0.0001
T3+T4	19(13.0)	127(87.0)	
N value			
N0	43(22.8)	146(77.2)	0.9455
N1+N2	26(22.4)	90(77.6)	
Distant metastasis			
M0	68(22.7)	232(77.3)	0.8876
M1	1(20.0)	4(80.0)	
LN metastasis			
No	43(22.8)	146(77.2)	0.9455
Yes	26(22.4)	90(77.6)	
Grade			
Well	7(15.2)	39(84.8)	0.4047
Moderate	61(24.2)	191(75.8)	
Poor	1(20.0)	4(80.0)	
Grade			
Well	7(15.2)	39(84.8)	0.1846
Moderate+poor	62(24.1)	195(75.9)	
Overall survival			
No	52(36.9)	89(63.1)	<0.0001
Yes	17(10.4)	147(89.6)	
PD-L1 expression			
Low	29(16.9)	143(83.1)	0.0062
High	40(30.1)	93(69.9)	

### OSCC patients with distant metastasis had high PD-L1 expression

**Fig 1:**
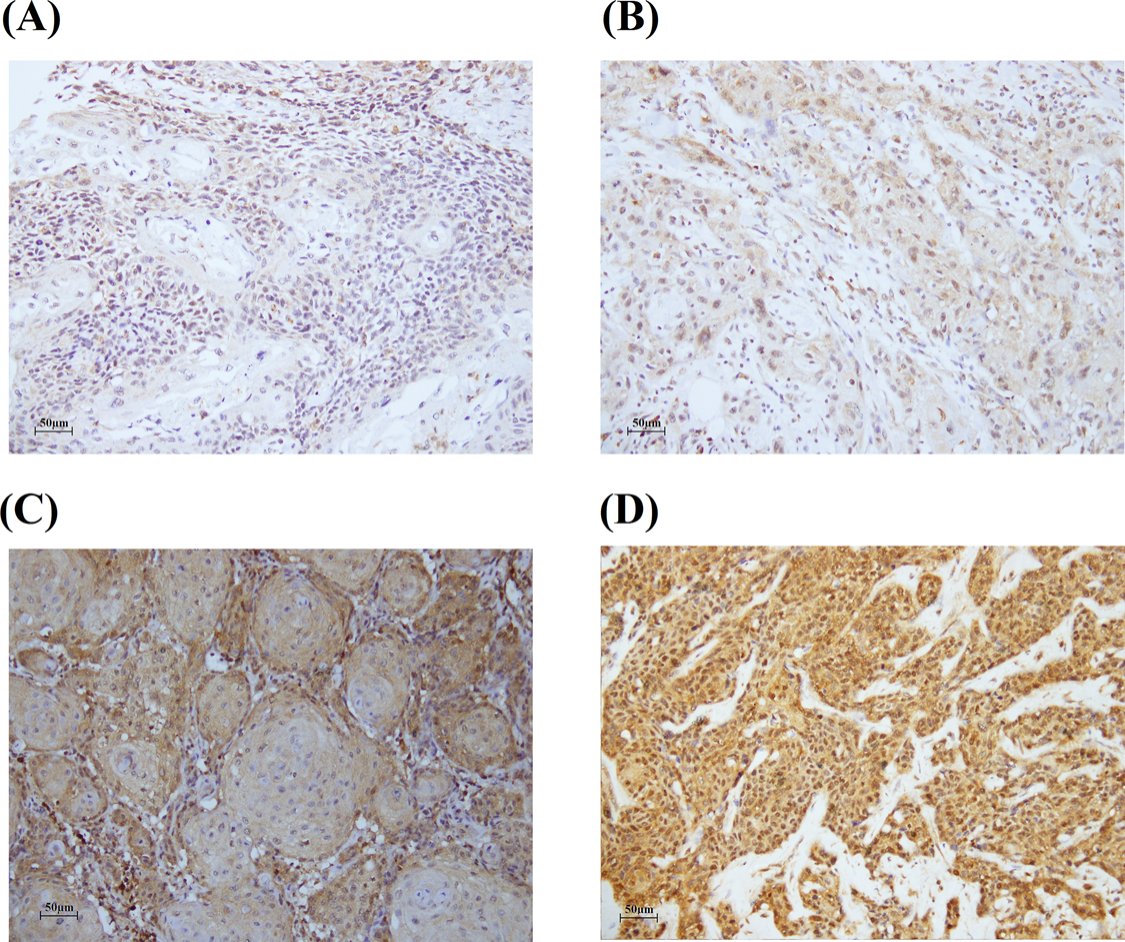
Representative immunostaining of PD-L1 in OSCC in tissue arrays. PD-L1 expression intensities were (A) 0; (B) 1; (C) 2; (D) 3.

PD-L1 expression was evaluated by IHC staining of tissue microarray sections (Fig 1). The cytoplasmic PD-L1 expression intensity was scored by two pathologists. High PD-L1-expression (staining intensity: 2–3) was significantly associated with distant metastasis (percentage of patients in M1 stage: 0.0% of patients with low PD-L1 vs. 3.8% of patients with high PD-L1, p = 0.0103, Table 2). However, PD-L1 expression had no significant association with age, smoking, betel quid chewing, alcohol consumption, tumor stage, T value, N value, or pathologic grading (Table 2). PD-L1 expression was also not associated with the death rate of patients in terms of overall survival (Table 2).

**Table 2:** The association between tumor PD-L1 expression and clinical parameters in 305 OSCC patients.

		PD-L1 expression		
Parameter	Case No.	Low (%)	High (%)	P value
Age				
<56	162	98(60.5)	64(39.5)	0.1243
≥56	143	74(51.7)	69(48.3)	
Gender				
Female	69	29(42.0)	40(58.0)	0.0062
Male	236	143(60.6)	93(39.4)	
Smoking				
No	142	78(54.9)	64(45.1)	0.6304
Yes	163	94(57.7)	69(42.3)	
Alcohol consumption				
No	167	98(58.7)	69(41.3)	0.5468
Yes	134	74(55.2)	60(44.8)	
Betel quid chewing				
No	165	91(55.2)	74(44.8)	0.5085
Yes	115	68(59.1)	47(40.9)	
Stage				
I+II	110	61(55.5)	49(44.5)	0.8039
III+IV	195	111(56.9)	84(43.1)	
T value				
T1+T2	159	94(59.1)	65(40.9)	0.3164
T3+T4	146	78(53.4)	68(46.6)	
N value				
N0	189	108(57.1)	81(42.9)	0.7362
N1+N2	116	64(55.2)	52(44.8)	
Distant metastasis				
M0	300	172(57.3)	128(42.7)	0.0103
M1	5	0(0.0)	5(100.0)	
Grade				
Well	46	29(63.0)	17(34.0)	0.3264
Moderate+poor	257	142(55.3)	115(44.7)	
Overall survival				
Alive	141	87(61.7)	54(38.3)	0.0830
Dead	164	85(51.8)	79(48.2)	

### Prognostic role of PD-L1 expression according to clinicopathological characteristics of oral cancer

**Fig 2:**
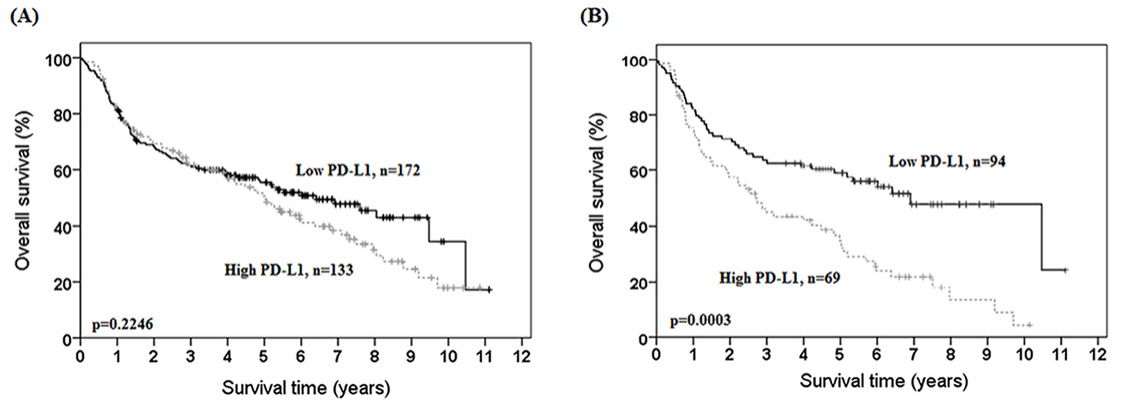
Kaplan-Meier actuarial analysis of PD-L1 expression in overall survival. (A) all patients and, (B) patients with smoking habits.

We also examined the potential prognostic role of PD-L1 expression in OSCC patients. Overall survival data were collected and no data were missing among 305 patients. We evaluated the prognostic role of clinicopathological characteristics and PD-L1 expression in OSCC patients by the Kaplan-Meier analysis and Cox regression model. The results of Kaplan-Meier analysis and univariate analysis of the influence of various parameters on overall survival are shown in Table 3 and Fig 2A. Male gender, smoking, and advanced stage were significantly associated with poor clinical outcome for univariate analysis (according to gender: HR = 2.740, 95% CI = 1.657–4.531, p<0.001; smoking: HR = 1.414, 95% CI = 1.033–1.934, p = 0.0305; stage: HR = 2.039, 95% CI = 1.441–2.886, p<0.001; T value: HR = 2.142, 95% CI = 1.563–2.937, p<0.001; N value: HR = 2.051, 95% CI = 1.498–2.809, p<0.001, Table 3). Distal metastasis and pathologic grading were not significantly associated, but these clinicopathological characteristics had a trend toward significance (Table 3). Factors of betel quid chewing, alcohol consumption and PD-L1 cytoplasm intensity were not significantly associated with prognosis in the univariate analysis (Table 3).

**Table 3:** Univariate analysis of the influence of various parameters on overall survival in OSCC patients.

		Univariate analysis		
Parameter	Category	HR	95% CI	P
Gender	Male/Female	2.740	1.657–4.531	< .0001
Smoking	Yes/ No	1.414	1.033–1.934	0.0305
Alcohol consumption	Yes/ No	1.253	0.918–1.711	0.1556
Betel quid chewing	Yes/ No	1.306	0.938–1.818	0.1140
Stage	III+IV/ I+II	2.039	1.441–2.886	< .0001
T value	T3+T4/ T1+T2	2.142	1.563–2.937	< .0001
N value	N1+N2/ N0	2.051	1.498–2.809	< .0001
Distant metastasis	M1/ M0	2.627	0.971–7.108	0.0573
Grade	Moderate+poor/ Well	1.602	0.993–2.586	0.0536
PD-L1 expression	High/Low	1.209	0.890–1.643	0.2254

We also investigated the prognostic role of PD-L1 in OSCC patients with different clinicopathological characteristics by analyzing the clinical outcome by multivariate analysis. Table 4 shows the multivariate analysis of the influence of various parameters on overall survival in OSCC patients. High PD-L1 expression was significantly associated with poor prognosis in male patients and smoking patients (in male: HR = 1.556, 95% CI = 1.124–2.153, p = 0.0077; in smoker: HR = 2.058, 95% CI = 1.376–3.077, p = 0.0004, Table 4). The median survival years for males with PD-L1 staining intensity 0-1/2-3 were 4.1/3.7 years. The median survival years for smokers with PD-L1 staining intensity 0-1/2-3 were 4.2/3.3 years (Fig 2B). PD-L1 could therefore be an independent prognostic marker in male patients and smokers, which was confirmed by multivariate analysis. This prognostic function of PD-L1 was therefore significant in patients with specific clinicopathological characteristics.

**Table 4:** Multivariate analysis of the influence of various parameters in PD-L1 on overall survival in OSCC patients.

Parameter	Case no. (PD-L1 Low/High)	Median Survival year	HR^a^	95% CI	P value
All cases	172/133	4.0/3.8	1.345	0.987–1.834	0.0609
Gender					
Female	29/40	3.7/4.3	0.534	0.204–1.397	0.2008
Male	143/93	4.1/3.7	1.556	1.124–2.153	0.0077
Smoking					
No	78/64	3.7/4.6	0.614	0.373–1.011	0.0552
Yes	94/69	4.2/3.3	2.058	1.376–3.077	0.0004
Stage					
I+II	61/49	4.8/4.7	1.252	0.692–2.265	0.4573
III+IV	111/84	3.6/3.4	1.243	0.868–1.780	0.2352
Grade					
Well	29/17	4.6/4.7	0.997	0.368–2.701	0.9959
Moderate+poor	142/115	3.8/3.8	1.192	0.859–1.653	0.2928

^a^gender, smoking, stage and grade were adjusted in multivariate analysis.

## Discussion

The purpose of this study was to analyze the clinicopathological characteristics according to gender and PD-L1 expression in a large series of OSCC samples and to provide what is, to the best of our knowledge, the first evaluation of the potential role of PD-L1 in clinical prognosis. This is the first study to show that PD-L1 expression, determined by IHC staining, could be an independent prognostic marker for male and smoker OSCC patients. Overexpression of PD-L1 has been identified in several cancers, including the head and neck [6,8,12,15,16,17,18,19,20], but the evidence for a prognostic role of PD-L1 in malignancies is inconsistent, although most studies reveal a worse outcome correlation [17,21,23,24]. Our findings indicated that a higher PD-L1 expression level was correlated with several clinicopathological factors, such as female patients and distant metastasis. We also found that PD-L1 could serve as an independent prognostic marker in male patients and smokers, as confirmed by multivariate analysis. This latter finding implies that the PD-L1 overexpression in OSCC tumors might be associated with OSCC tumor progression in patients with specific clinicopathological characteristics.

Currently, the majority of OSCC patients with recurrence will not have any option for salvage surgery or radiation. Palliative therapy, including chemotherapy, which is usually platinum based, and traditional anti-tumor monoclonal antibodies (mAbs, e.g. cetuximab) function by targeting molecules on the tumor cell surface (e.g. EGFR), resulting in functional receptor blockade and inhibition of signal transduction [32,33,34]. Unfortunately, the median survival for patients with loco-regionally recurrent or metastatic disease, treated with palliative chemotherapy alone, is only 8 to 10 months [32,33,34]. Therefore, new therapeutic options are vitally needed for these patients.

The T-cells play a dominant role in host antitumor immune function due to their capability to recognize cancer cells as abnormal and by further generation of a population of cytotoxic T lymphocytes that can infiltrate the tumor mass and kill tumor cells [7,9]. In recent years, many studies have confirmed that cancer cells can evade host immune systems by expressing certain ligands that down-regulate cytotoxic T lymphocytes through inhibitory pathways that are usually initiated by ligand-receptor interactions [9,11,14,17,30,31]. Thus, a new class of mAbs has emerged recently that is not necessarily designed to directly target the tumor, but is instead engineered to block or activate specific co-signaling pathways, resulting in enhanced anti-tumor immunity [5,7,11,14].

One of the most promising pathways for manipulation involves PD-L1, where its up-regulated expression in solid tumors provides direct tumor protection as well as reducing the activity of PDCD1 expressing tumor-infiltrating effector CD4 and CD8 T cells [5,7,11,14]. Initial phase I trials with anti PDCD1 or anti-PD-L1 mAbs have shown considerable promise, with response rates of 18–28% and 10–17%, respectively, in patients with advanced pre-treated melanoma, non-small cell lung cancer (NSCLC), and renal cell carcinoma [35]. These early clinical studies have now evolved into phase III trials in melanoma and NSCLC, and phase I trials in multiple other solid tumor types, including in patients with recurrent/metastatic squamous cell carcinoma of the head and neck, hepatocellular carcinoma, ovarian cancer, colorectal cancer, gastroesophageal adenocarcinoma, triple negative breast cancer, glioblastoma multiforme, and pancreatic cancer [36,37]. The ongoing clinical studies of PD-L1 and PDCD1 blockade in OSCC for better activation of the immune system might therefore show potential for further increasing therapeutic efficacy [38,39].

Our study has some limitations, including the regional source of our cases. The limitations of tissue microarrays also mean that the tissue cores cannot represent the whole tumor condition (45 cases were evaluated with three cores and 260 cases were evaluated with one core). Second, only overall survival but no relapse-free survival nor disease-free survival was investigated in this study. Third, the underlying mechanism of sex and smoking history contribute to the prognostic role of PD-L1 is not clear. Thus, more complete studies are still needed in the future. However, our results suggested that patients with high PD-L1 expression had poor clinical outcome and might require PD-L1-targeted immunotherapy to improve their prognosis.

## Supporting Information

S1 Fig Representative immunostaining of PD-L1. (A) tonsil; (B) non-neoplastic squamous epithelium.
(JPG)
