# Supplementary figures and images for: High PD-L1 Expression Correlates with Metastasis and Poor Prognosis in Oral Squamous Cell Carcinoma
Source: PLoS One. 2015 Nov 12;10(11):e0142656. doi: 10.1371/journal.pone.0142656 (PMC4642967; doi:10.1371/journal.pone.0142656)

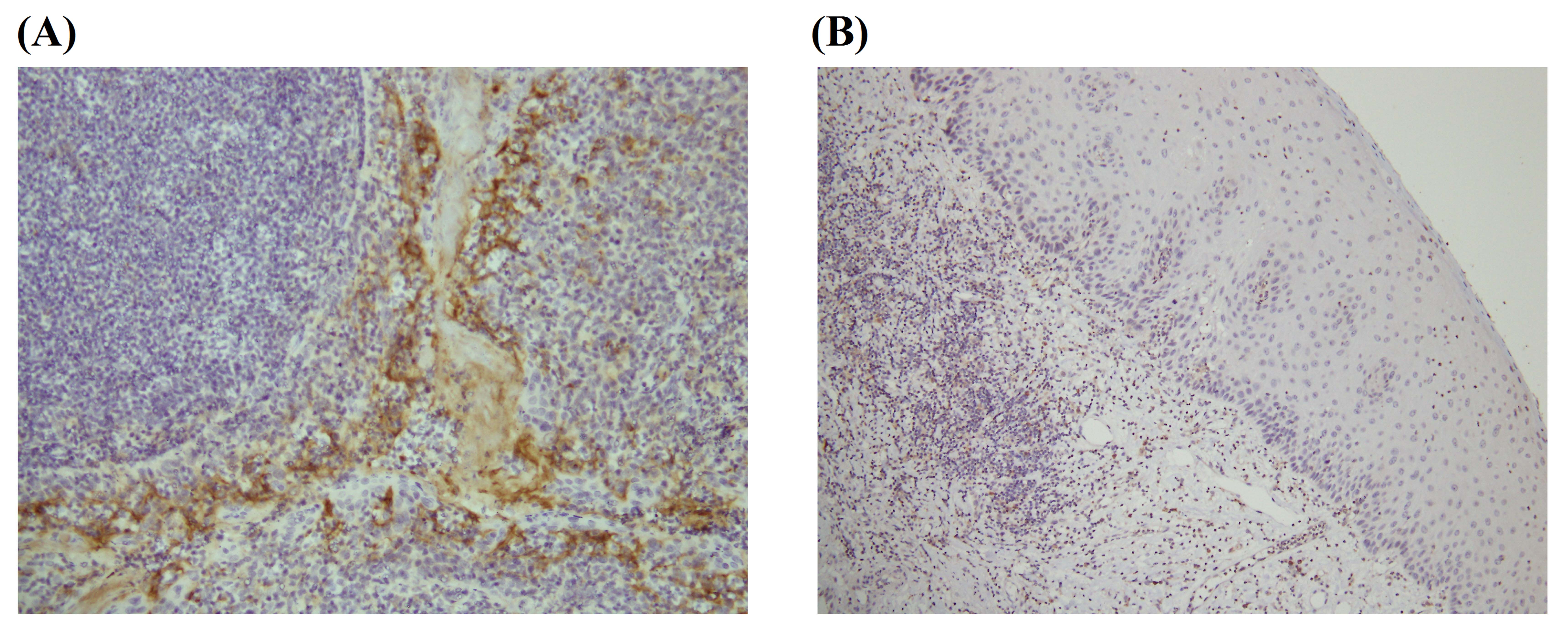

Supplement: S1 Fig — (A) tonsil; (B) non-neoplastic squamous epithelium. (JPG) [file pone.0142656.s001.jpg]
